# Strain FBG-Based Sensor for Detecting Fence Intruders Using Machine Learning and Adaptive Thresholding

**DOI:** 10.3390/s23115015

**Published:** 2023-05-24

**Authors:** Ahmad Elleathy, Faris Alhumaidan, Mohammed Alqahtani, Ahmed S. Almaiman, Amr M. Ragheb, Ahmed B. Ibrahim, Jameel Ali, Maged A. Esmail, Saleh A. Alshebeili

**Affiliations:** 1 Electrical Engineering Department, King Saud University, Riyadh 11421, Saudi Arabiajhasan.c@ksu.edu.sa (J.A.);; 2 KACST-TIC in Radio Frequency and Photonics (RFTONICS), King Saud University, Riyadh 11421, Saudi Arabia; 3 Smart Systems Engineering Laboratory, Communications and Networks Engineering Department, Faculty of Engineering, Prince Sultan University, Riyadh 11586, Saudi Arabia

**Keywords:** fiber Bragg grating, optical sensing, adaptive thresholding, linear discriminant analysis, logistic regression, machine learning

## Abstract

This paper demonstrates an intruder detection system using a strain-based optical fiber Bragg grating (FBG), machine learning (ML), and adaptive thresholding to classify the intruder as no intruder, intruder, or wind at low levels of signal-to-noise ratio. We demonstrate the intruder detection system using a portion of a real fence manufactured and installed around one of the engineering college’s gardens at King Saud University. The experimental results show that adaptive thresholding can help improve the performance of machine learning classifiers, such as linear discriminant analysis (LDA) or logistic regression algorithms in identifying an intruder’s existence at low optical signal-to-noise ratio (OSNR) scenarios. The proposed method can achieve an average accuracy of 99.17% when the OSNR level is <0.5 dB.

## 1. Introduction

In recent years, optical fibers have enabled the innovation of several technologies in the field of sensing. This is because the optical fiber sensors have minimal transmission loss, immunity to electromagnetic interference, passive operation, high sensitivity, and reliability in harsh conditions, making them of greater importance for sensing [1]. Optical fiber sensors can measure different physical parameters, such as temperature, strain, or pressure [2,3]. The sensing is achieved when the properties of a propagating light wave, such as intensity, phase, polarization, or wavelength, are modulated by the physical parameters [4]. Different methods for optical fiber sensing have been explored in the literature, such as reflectometry-based sensors and interferometry-based sensors. Grating-based sensors (e.g., fiber Bragg grating (FBG)) are considered point or multi-point sensors (quasi-distributed) [4]. Compared to the reflectometry-based sensors and interferometry-based sensors, FBG-based sensing has several advantages: (i) it has predetermined locating capabilities and can be assigned to either effective sensing or non-sensing fiber segments, (ii) it responds linearly to the impact of external events, and (iii) it has a higher SNR than other distributed sensing techniques [5]. However, event identification in optical sensing systems remains a challenging problem in practical conditions due to environmental noise and interfering events.

Optical sensing systems for fenced perimeter security comprise three primary components: an optical sensing system, a feature extraction method, and a classification algorithm [6]. The optical sensing system is responsible for capturing signals from the perimeter, including the presence of potential intruders or environmental noise, such as wind. Subsequently, the feature extraction method analyzes the captured data to extract relevant features, such as potential intruders or stable environmental conditions. Finally, the classification algorithm utilizes these features to accurately identify potential threats or intruders, distinguishing, for example, between human movement and environmental noise. By combining these components, the security system can quickly detect and respond to potential threats, making it favorable for various applications ranging from protecting critical infrastructure to protecting private properties.

The interferometry-based sensing systems, such as the dual Mach–Zehnder interferometer (DMZI) and Sagnac interferometer, were utilized for event detection in fenced perimeter security systems [7,8,9,10,11]. In Ref. [7], the proposed intruder pattern recognition system utilized the DMZI structure and a pre-processing method based on an empirical mode decomposition (EMD) to extract features and feed them to a radial basis function (RBF) neural network as a classifier network. Here, EMD is first used as a pre-processing step to separate event signals into intrinsic mode functions (IMFs). Then, the kurtosis characteristic was taken out, and the RBF neural network was used to classify the data. The experimental results showed that the proposed method achieved an average recognition rate of over 85.75% for four types of human activities detected on the fence. The EMD decomposition results can differ depending on the used parameters. In Ref. [8], a deep metric-learning network combined with recurrent plot (RP) coding was proposed to improve the accuracy of target event recognition in an open environment with unknown events, where the DMZI was used in the perimeter sensing system. In Ref. [9], an intrusion event recognition scheme based on a convolutional prototype network (CPL) was proposed. The proposed method enabled end-to-end feature extraction and recognition by integrating relevant variables of prototype learning into the training process of a multiscale convolutional neural network (MSCNN) as trainable parameters. In Ref. [10], recurrent plot (RP) and deep learning methods were used to detect abnormal events. By encoding the sensing signals into two-dimensional images using the RP algorithm, the inception network extracted features from these images to identify the intruder’s signal. In Ref. [11], the proposed intruder pattern recognition algorithm utilized the Sagnac interferometer structure and consisted of pre-processing and pattern recognition using multi-layer perceptron neural networks (MLP-NNs). The power spectrum of the vibration signal was used in the pre-processing step to extract relevant features, and a selected frequency band of 0 Hz to 2000 Hz was used to construct a frequency sequence. The frequency sequence was serialized and used as input for the MLP-NN model, classifying the pattern into three categories: normal situation, intrusion events, and interference. Furthermore, a comprehensive review of recent backscattered sensing (phase-OTDR) developments for perimeter security monitoring systems was presented in [6].

As quasi-distrusted sensors, FBGs have attracted much interest in the applications of strain and temperature measurements for highway structures, buildings, railways, and gesture recognition [12,13,14,15,16,17]. The FBG-based sensing systems work by measuring the displacement of the Bragg peak in the spectrum and then deriving the change in the physical quantity. Conventional optical FBG sensing systems incorporated wavelength peak detection algorithms for that purpose [18,19]. However, these methods needed a high SNR and side lobe suppression on the demodulated spectrum. A growing interest has been in integrating machine learning (ML) techniques into optical FBG sensing systems. For example, some researchers investigated using neural networks for peak tracking [20,21,22,23]. Additionally, other researchers recently investigated using machine learning algorithms with the optical FBG sensors for leakage detection, subway track vibration sensing, liquid level estimation, and temperature sensing [24,25,26,27].

One important application for optical FBG sensors is to detect any intruder in fenced perimeter security applications [28,29]. Previous works demonstrated installing optical FBG sensors on a fence around the premises, where the sensing system (i.e., optical interrogator) notifies when the intruder causes the autocorrelation to exceed a threshold [30], or after the sensing system compares the reflected signal with different intruder patterns [31]. Moreover, in [32], an FBG-based sensing system was installed on a fence, and the feature data were extracted by the principal component analysis (PCA), which were then used to identify the event with a K-nearest neighbor classifier. However, an investigation of the benefits of using machine learning algorithms in classifying the intruder in noisy-based sensing scenarios is missing, where machine learning could be beneficial to enhance the identification of intruders in noisy signals [33,34].

In this paper, we propose simple algorithms for intruder detection and event classification at low optical signal-to-noise ratio (OSNR) scenarios. In particular, we (i) experimentally install an FBG sensor on a fence outside the College of Engineering building at King Saud University and connect the fence to the intruder detection system through 50 km of fiber, (ii) attenuate the interrogator signal until the OSNR level is lower than 0.5 dB, where detecting the peak becomes challenging, (iii) use linear discriminant analysis (LDA) and logistic regression algorithms in the ML models to identify the class of an intruder as no intruder, intruder, or wind, and (iv) investigate the performance of the intruder detection system when the ML models incorporate or do not incorporate adaptive thresholding for peak detection as a pre-processing stage. As an example of the adaptive thresholding algorithms, we use the short-term average/long-term average (STA/LTA) algorithm [35]. The experimental results show that the machine learning performance can be improved when the STA/LTA adaptive thresholding is incorporated, and the accuracy could reach up to 99.17%.

The paper is organized as follows. Section 2 presents the concept and background of the used FBG, the machine learning algorithms (including LDA and logistic regression classifiers), and the STA/LTA peak detection algorithm. Section 3 presents the experimental setup and data acquisition. Section 4 presents the data separation and verification. In Section 5, we discuss the results. Finally, we provide concluding remarks in Section 6.

## 2. Concept and Background

### 2.1. Concept

**Figure 1:**
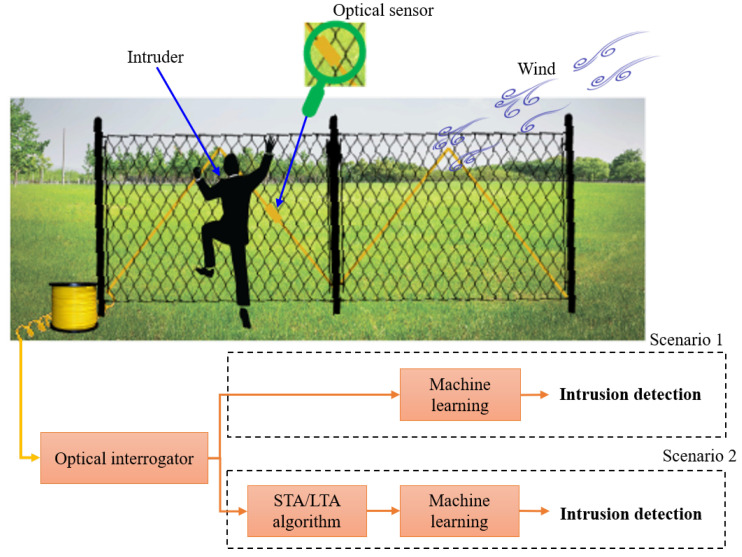
The concept of fence intruder detection using a strain FBG-based sensor, adaptive thresholding, and machine learning.

The concept of our paper is presented in Figure 1. An intruder is assumed to climb a fence wired with an FBG sensor. The FBG receives the light from the optical interrogator and reflects the light at a specific wavelength. The optical interrogator receives the reflected light and processes it. For example, for the “no intruder” conditions, the FBG will reflect the light at its specified Bragg wavelengths. However, when other conditions around the FBG occur (such as the intruder or wind), the Bragg wavelength shifts in the recorded optical spectrum in the optical interrogator. Here, we investigate two scenarios. In the first scenario, we train the machine learning models (i.e., the LDA algorithm or logistic regression) in the interrogator to identify the class of the intruder directly using the light signal reflected from the optical FBG. In the second scenario, we add the STA/LTA algorithm as a pre-processing stage for peak detection before the intruder detection ML models in the interrogator. In both scenarios, we train the ML models to classify the intruder as either: (i) no intruder, (ii) intruder, or (iii) wind. Ultimately, we compare the machine learning performance in the two scenarios and show the advantages of using the STA/LTA algorithm for peak detection as a pre-processing step for the machine learning model.

### 2.2. The Fiber Bragg Grating Sensor

The FBG is a type of optical fiber sensor that is written on a short segment of a fiber. In the FBG sensor, the refractive index of the fiber’s core is periodically modified at a specific pitch [12]. When an FBG is exposed to a broadband light source, the FBG will reflect only the wavelengths of light that correspond to the Bragg wavelength ($\lambda_{B}$). All other wavelengths of light will pass through the FBG without being reflected. Any change in the physical environment surrounding the fiber will make the FBG characteristics, such as the refractive index ($n_{e}$) or grating pitch (Λ), change, which affects the Bragg reflection wavelength allowing to sense the physical effects around the FBG. $\lambda_{B}$ can be expressed as in Equation (1):

(1) $$\lambda_{B}=\;2n_{e}\Lambda$$

When the FBG is used to sense its surrounding, any change in strain or temperature causes a shift in the Bragg wavelength as in Equation (2):

(2) $$\frac{\Delta \lambda_{B}}{\lambda_{B}}=\;k_{e}\varepsilon +{\Delta K}_{T}$$

where $K_{e}$ and $K_{T}$ are the strain and temperature coefficients of the FBG sensor, while ε is the engineering normal strain.

### 2.3. Machine Learning Algorithms

**Figure 2:**
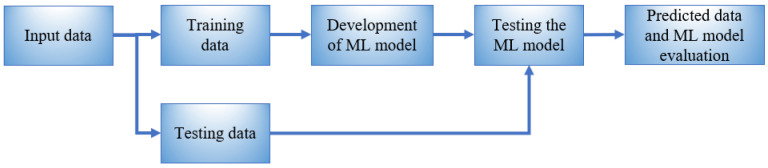
The processes for the ML model development.

In our work, we aim to build an ML model that can identify the outdoor event among the three classes using the reflected waveform from the FBG sensor and label the output to one of the classes shown in Table 1. Figure 2 shows the standard ML model design processes. The processes start with splitting the input data into training and testing data. The training data are used to develop the ML model. The developed ML model is then tested using the testing data [36,37,38]. Finally, the model’s robustness is assessed from its predicted output data. We will investigate two machine learning algorithms for classification, which are the linear discriminant analysis (LDA) algorithm and the logistic regression algorithm. The linear discriminant analysis (LDA) and logistic regression are supervised learning algorithms that have recently been used in different fields, such as the classification of oil slicks and look-alike slicks [39], breast cancer diagnosis [40], classification of dyadic conversation scenarios [41], power quality disturbances [42], and seizure detection [43]. We opt to explore these three algorithms for intruder detection because of their simplicity and low computational cost.

#### 2.3.1. Linear Discriminant Analysis

The first algorithm we will investigate in the ML model to classify the input signal to one of our three classes is the linear discriminant analysis algorithm [44]. The LDA algorithm divides the data from different classes into groups, with all samples of the same group sharing the same mean with different variances. It does so by maximizing Fisher’s criterion [45]. The Fisher criterion is used to project the whole data from a higher-dimensional space to a lower-dimensional space so that a separating line can be drawn between the data classes. Therefore, maximizing Fisher’s criterion maximizes the distance between the centered means of different data groups and minimizes the scattering within the same group. To classify data samples of two different classes, the LDA model is trained to maximize Fisher’s criterion, which is defined by [46]

(3) $$F\left(\alpha \right)=\frac{\alpha^{T}\mu_{1}-\alpha^{T}\mu_{2}}{\alpha^{T}C\alpha }$$

where $\mu_{1}$, and $\mu_{2}$ are the means of data of the two classes, *C* is the common covariance matrix of the dataset, and α is a vector of linear coefficients that is required to maximize the Fisher’s factor such that $\alpha =[\alpha_{1},\alpha_{1},\cdots ,\alpha_{n}]$. After training the LDA-based ML classifier using a dataset of two classes $K_{1}$ and $K_{2}$, we can map a new testing sample *x* to class $K_{1}$ by [47]

(4) $$\alpha^{T}\left(x-\left(\frac{\mu_{1}+\mu_{2}}{2}\right)\right)>\log\frac{p(K_{1})}{p(K_{2})}$$

where $p(K_{1})$ is the probability of class $K_{1}$, and $p(K_{2})$ is the probability of class $K_{2}$.

#### 2.3.2. Logistic Regression

**Figure 3:**
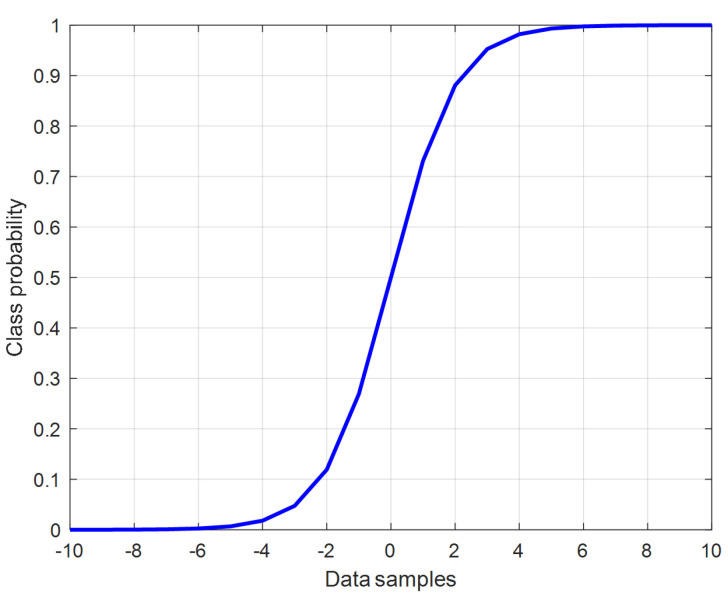
The standard sigmoid function.

The second algorithm we will investigate in the ML model to classify the input signal to one of our three classes is logistic regression. Logistic regression is used in binary classification problems to distinguish between data of two different classes. However, it shares the mathematical formula of linear regression, which is given by [48]

(5) $$z=\sum_{i=1}^{n}w_{i}x_{i}+b$$

where $x_{i}$ is the data sample, $w_{i}$ is the weight coefficient acquired through the training process, *n* is the length of the feature vector, and *b* is the intercept. Logistic regression builds upon linear regression by using the output of Equation (5) as an input to the standard logistic (sigmoid) function as follows [49]:

(6) $$p\left(z\right)=\frac{1}{1+e^{-z}}$$

where p(z) is the classification probability such that the given data sample *z* is classified to class ‘zero’ if p(z) is close to zero; otherwise, it is classified to class ‘one’. Figure 3 shows the standard shape of the sigmoid function.

### 2.4. The Short-Term Average/Long-Term Average (STA/LTA) Algorithm

The STA/LTA algorithm is an adaptive thresholding algorithm that can detect a peak in noisy environments. For example, the STA/LTA algorithm has been used for peak and anomaly detection applications, such as detecting seismic signals in geophysics [35,50]. It does so by calculating the ratio of the average energy in a short-term leading window to that in a long-term trailing window of a signal. In our work, we aim to use the STA/LTA algorithm as a pre-processing function before applying the ML processes (i.e., linear discriminant analysis (LDA) or logistic regression algorithms) such that the output data of the STA/LTA algorithm become the input to the ML algorithm.

**Figure 4:**
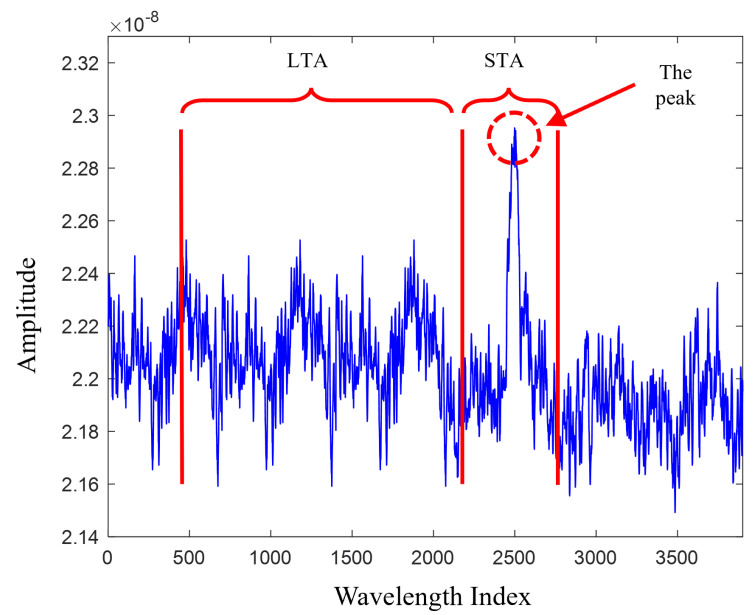
The STA/LTA calculation for finding the peak of a signal in a noisy environment. The peak is detected when the ratio of the average energy in a short-term leading window to the average energy in a long-term trailing window of a signal exceeds a certain threshold.

**Figure 5:**
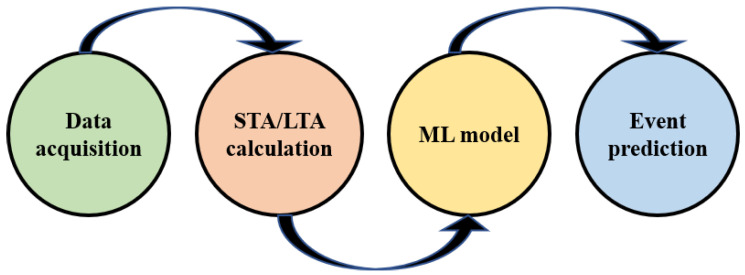
The flow chart of integrating the STA/LTA algorithm and the ML models.

Figure 4 demonstrates identifying a peak in a noisy environment using the STA/LTA algorithm. Here, an event is present if the STA/LTA ratio (η) is higher than a predefined threshold (α), where η can be calculated in the following way: [50]:

(7) $$\eta =\left\{\begin{array}{c} 0\;\;if\;\;\frac{\sum_{i=n-S+1}^{n}{\left|x_{i}\right|}^{2}}{\sum_{i=n-L-S+1}^{n-S}{\left|x_{i}\right|}^{2}}<\alpha \\ 1\;\;if\;\;\frac{\sum_{i=n-S+1}^{n}{\left|x_{i}\right|}^{2}}{\sum_{i=n-L-S+1}^{n-S}{\left|x_{i}\right|}^{2}}\geq \alpha \end{array}\right.$$

where $x_{i}$ is the *i*th sample of the digitized signal, *S* is the length of the STA window, and *L* is the length of the LTA window. In this study, we set the size of the short window to one sample and the size of the long window to 100 samples. The parameter α is a coefficient computed using background noise to obtain a certain false alarm probability ($P_{fa}$) that maximizes the probability of detection ($P_{d}$). Figure 5 outlines the flow chart of the proposed integration between the STA/LTA algorithm and ML algorithms.

## 3. Experimental Setup and Data Acquisition

### 3.1. Experiment Setup

**Figure 6:**
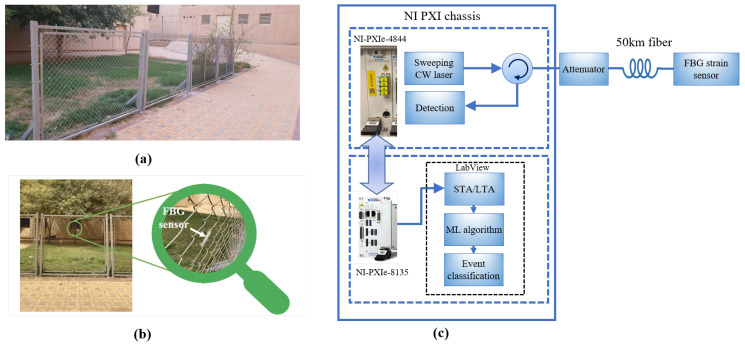
(**a**) The installed fence at King Saud University to conduct the experiment. (**b**) Attaching the FBG sensor to the fence. (**c**) The experiment setup.

**Figure 7:**
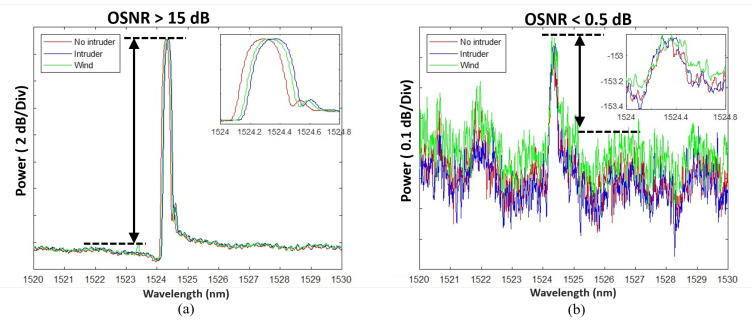
Examples of waveforms for each class: (**a**) the recorded waveform for the FBG sensor placed on the fence through 50 km of fiber from the interrogator showing higher than 15 dB of OSNR, (**b**) the recorded data after adding the attenuator on the same setup to lower the OSNR below 0.5 dB.

To build a training and testing dataset that helps in building the machine learning model, an outdoor fence was installed for this purpose. Figure 6a shows the installed fence at King Saud University, which is made of metal. The designed fence has dimensions of 8.8 m (length) × 1.7 m (height). The fence properties are presented in Table 2. The FBG sensor was attached to the fence as shown in Figure 6b using Scotch Magic Tape so that the FBG sensor can sense any vibration in the fence due to wind or climbing. To have a remote sensing system, the interrogator (PXIe-4844) was kept inside the lab connected to the FBG sensor through a 50 km of single-mode fiber (SMF-28) as shown in the experiment setup in Figure 6c. The FBG is also written on SMF-28 fiber. The interrogator is a module that transmits the light from a sweeping laser source and detects the reflections. The reflected light for the three scenarios at a distance of 50 km is presented in Figure 7a, where the wavelength shift could be observed with >15 dB of OSNR. In order to emulate the lower OSNR scenarios, we add an optical attenuator and adjust the OSNR level to lower than 0.5 dB as shown in Figure 7b. Here, it becomes obvious that conventional peak detection algorithm may not work well, leading to false alarms or missed events, and machine learning tools may help identify the intruder class by analyzing the features of the interrogator signal. Table 3 and Table 4 show the characteristics of the optical interrogator and the FBG sensor (OS3100) [51], respectively.

### 3.2. Data Acquisition

We acquired the data as follows. The PXIe-4844 transmits a sweeping CW laser light in the 1510–1590 nm range. The FBG is designed with a Bragg wavelength at 1524.3 nm, reflecting the light at the Bragg wavelength back to the transmitter. When the signal reaches the PXIe-4844 module, it starts recording the signal at a rate of 10 Hz. The data are then transferred to the embedded controller NI-PXIe-8135.

We emulate the intruder class using a person climbing the fence and emulate the wind class using an air blower. Examples of the collected waveform for each class are shown in Figure 7b. Every record consists of 2501 data points for representing the optical spectrum over the range 1520–1530 nm. We construct our dataset out of 134 records as 50 records of the no-intruder class, 34 records of the intruder class, and 50 records of the wind class. The intruder records are collected when a person standing next to the fence starts pulling it. The intruder remains in the same position for a few seconds, which is the time we use to collect the persistent readings. In terms of the wind, we use an air blower with the specifications presented in Table 5 that is targeted to the FBG sensor on the fences to cause enough stress to imprint a signature on the interrogator optical spectrum waveform. All the records are taken over a short duration to ensure consistency.

## 4. Data Separation Verification

In this section, we accumulate all the recorded data for numerical analysis and to verify the applicability of our collected data for machine learning classification using the LDA and logistic regression algorithms by investigating (i) whether the data follow normal distribution using the quantile–quantile plots (Q-Q plots), and (ii) the separability of the data using the t-distribution stochastic neighbor embedding (t-SNE) algorithm.

The Q-Q plot compares two probability distributions by plotting their quantiles against each other [52]. The distributions are identical if the scatter points lie on a straight line in the plot; otherwise, they are not identical. In this work, we draw the Q-Q plot for the recorded data of the different events against the normal distribution.

On the other hand, the t-SNE is a visualization and exploration algorithm that projects data samples from a higher-dimensional space to a vector of two points, each plotted in a plane, to facilitate the visualization of data samples in a 2D space [53,54]. The t-SNE algorithm provides a simple visualization sense compared to other visualization methods, such as box-plot or histograms, which require some statistical background to analyze the plots. This algorithm computes similarity measures between pairs of instances in the high- and low-dimensional spaces. The obtained measures are then optimized using a cost function. The t-SNE algorithm has been used in many applications, such as biomedical fields, genomics, and computer security [55]. The t-SNE algorithm plot displays the data as clusters in 2D. Each cluster represents one of the different classes in the problem. The classes can be classified easily if the clusters are separable, i.e., not overlapped. If overlapped, it is difficult to classify them with high accuracy.

**Figure 8:**
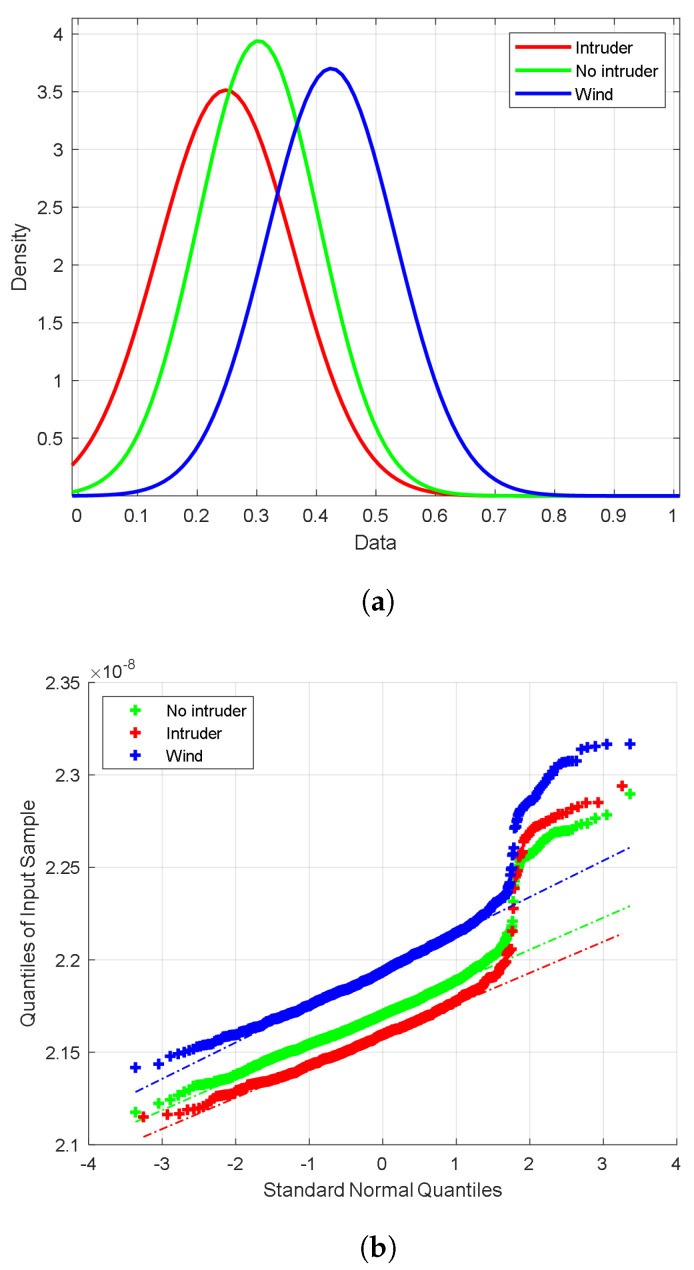

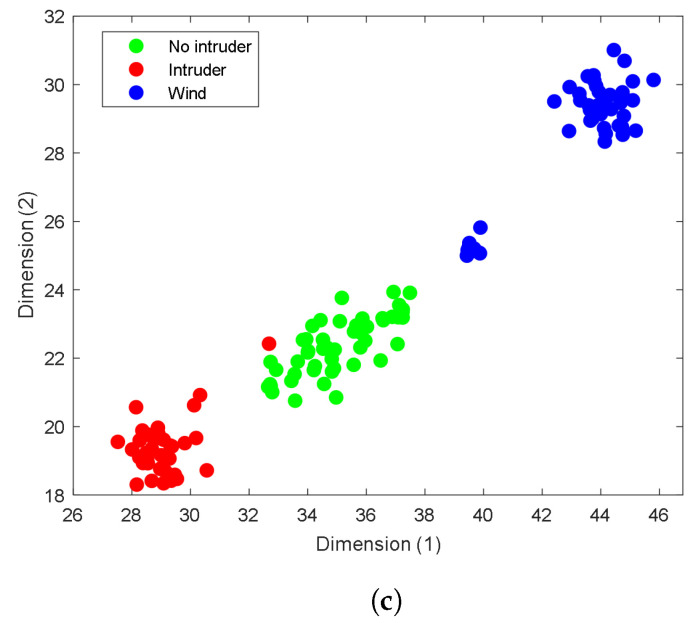
(**a**) The distribution of the collected dataset. (**b**) The Q-Q plots of the three classes. (**c**) The t-SNE plot of the collected data.

Figure 8a shows the statistical distribution of the data records. The x-axis (data axis) represents the values of data after normalization, while the y-axis (density axis) presents the proportion of these values. In Figure 8b, we show the Q-Q plots of the three classes. The figure clearly shows that the majority of the data (++ plot) of the three classes follow the theoretical quantiles (− plot), which means that the majority of the data follow a normal distribution; however, the higher values of the data (to the right) deviate slightly from the linear plot showing skewness in the data. This skewness is confirmed by the long right tail of the distributions of Figure 8a. Additionally, from Figure 8a,b, it is obvious that there is a linear shift between the distributions of the three data classes. Since the Q-Q plot curves are separable, the classifiers can work well with high classification accuracy.

Further, we apply the t-SNE algorithm to the data. Figure 8c shows the t-SNE plot, indicating that the data of the three classes are linearly separable. These results indicate that the three classes can be accurately classified correctly. Therefore, the results from the data verification using Q-Q and t-SNE plots indicate that linear classification algorithms, such as the LDA or logistic regression, can classify the different classes in this problem under consideration with high accuracy.

## 5. Results and Discussions

As mentioned above, we investigate two scenarios and compare their performances. In the first scenario, we train the machine learning models (i.e., the LDA algorithm or logistic regression) in the interrogator to identify the class of the intruder directly using the light signal reflected from the optical FBG. In the second scenario, we add the STA/LTA algorithm as a pre-processing stage for peak detection before the intruder detection ML model in the interrogator. The obtained results for these scenarios are as follows.

### 5.1. First Scenario: Directly Using the ML Models

**Figure 9:**
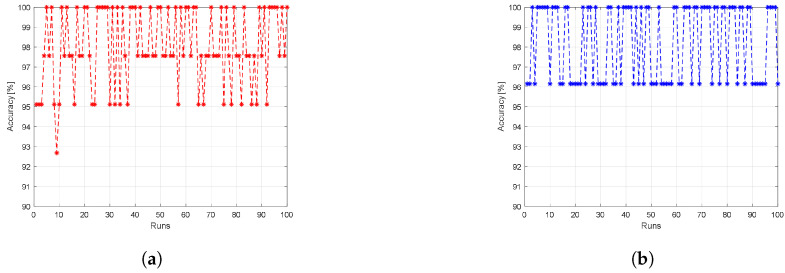
The achieved accuracy results when using raw data as input to (**a**) the LDA ML model and (**b**) the logistic regression ML model.

In the first scenario, we train the LDA and the logistic regression ML models using raw data from the experimentally collected dataset. We scale the entire dataset to be within the range of [0, 1] before training the LDA and the logistic regression models. We use 70% of the dataset records for training, while the other 30% is used for testing. The results are averaged over 100 independent runs, where for each run, the training and testing samples are reselected randomly, and thus we train a new ML model using different training data samples to ensure that the trained model is not biased to any subset of the dataset. Figure 9a displays the achieved average accuracy using the LDA model, while Figure 9b illustrates the achieved average accuracy using the logistic regression model for each run. In the LDA ML model, almost all the runs have a classification accuracy of more than 95%. On the other side, the accuracy for the logistic regression model is above 96% for all runs. Therefore, the logistic regression model has better classification performance than the LDA ML model. On average, the LDA classification model achieves 98% classification accuracy, while the logistic regression classifier model achieves 98.19%, slightly better than the LDA model.

### 5.2. Second Scenario: Using the STA/LTA Algorithm as a Pre-Processing Function before the ML Models

**Figure 10:**
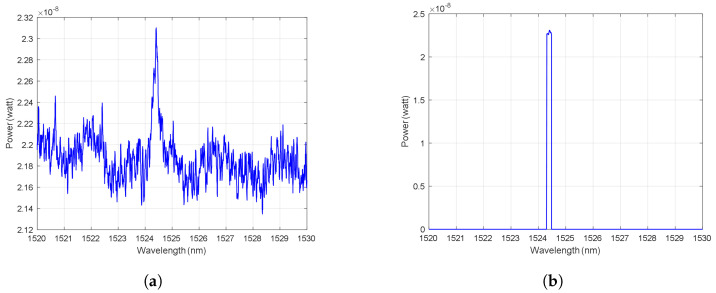
(**a**) An example of the optical interrogator recorded waveform. (**b**) The corresponding STA/LTA output.

**Figure 11:**
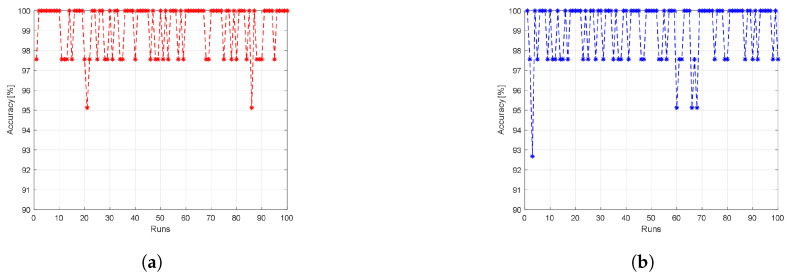
The achieved accuracy results when using the STA/LTA algorithm as a pre-processing function before (**a**) the LDA ML model, or (**b**) the logistic regression ML model.

In the second scenario, we add the STA/LTA algorithm to the LDA and logistic regression ML models as a pre-processing stage. In the STA/LTA algorithm, we set the long window to 100 samples and the short window to one sample. The value of α is set to be 1.028 to maintain the probability of a false alarm equal to 0.0224. The data before and after the STA/LTA algorithm are shown in Figure 10 for an arbitrary interrogator recorded signal. We notice that the STA/LTA algorithm can accurately extract the noisy signal peak. After peak detection using the STA/LTA algorithm, the reflected signal is cropped around the detected peak to form a window of size of 400 data points. Similar to the first scenario, we use 70% of the pre-processing dataset records for training the models, while the other 30% is used for testing. The results are averaged over 100 independent runs, where for each run, we train a new ML model using different and randomly selected data samples to ensure that the trained model is not biased to any subset of the dataset. The resulting accuracy of the LDA model is shown in Figure 11a, while Figure 11b depicts the resulting accuracy of the logistic regression model. The STA/LTA algorithm enhances the results and boosts the steadiness of the ML classification model, where most of the classification results now have values greater than 97% with an average accuracy over the 100 independent runs of 99.17% and 99% for the LDA and logistic regression classifiers, respectively. We note that because we use a new ML model for every run, we observe some variations in the results.

**Figure 12:**
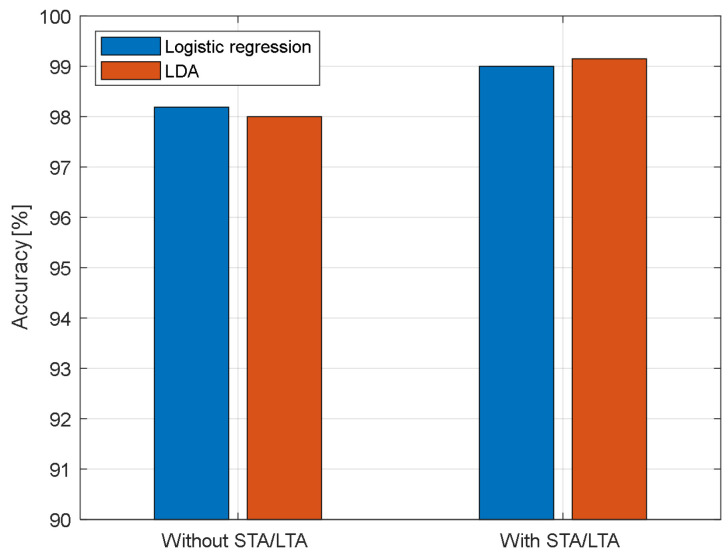
Comparison of the achieved results of the LDA and logistic regression classifiers with/without the STA/LTA algorithm.

**Figure 13:**
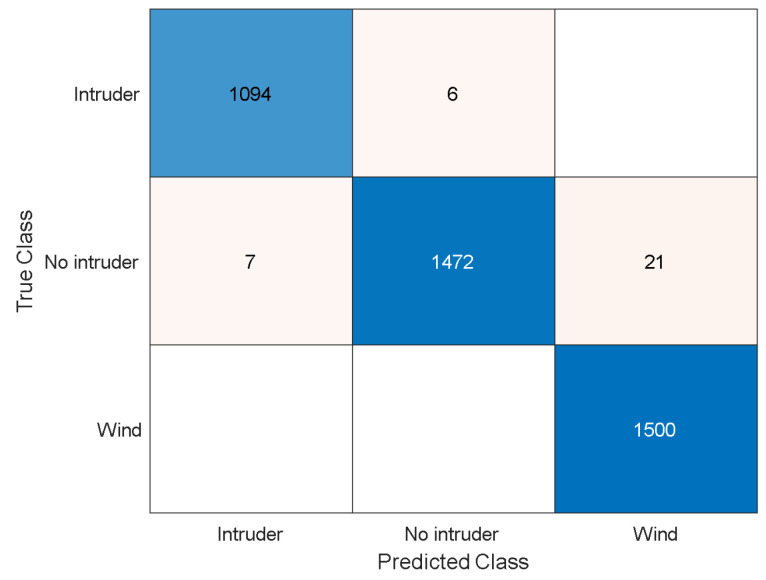
Confusion matrix of accumulated 100 independent runs of STA/LTA-LDA classifier.

Finally, in Figure 12, we compare the achieved results of LDA and logistic regression classifiers with and without the STA/LTA algorithm. Additionally, in Figure 13, we present the confusion matrix of accumulated results of 100 independent runs of STA/LTA-LDA classifier. It is clearly shown that the “No intruder” class has the most misclassified samples, as those samples are perplexed with samples of the “Wind” class; some samples of the “intruder” class are confused with samples of the “No intruder” class, while all samples of the “Wind” class are correctly classified over 100 independent runs. Our results indicate that the three classes can be accurately classified correctly at low OSNR even for the small dataset of 134 records.

## 6. Conclusions

In this work, an intrusion detection system using optical FBG sensors is proposed. The system exploits ML techniques to improve detection accuracy in fenced perimeter security applications under low OSNR conditions. To investigate the performance of the proposed system, we experimentally demonstrated installing the system on an outdoor fence. The experiments were conducted on the outdoor fence, considering three conditions: no intrusion, intrusion, and wind. The LDA and logistic regression algorithms were used as ML algorithms for developing classification models. The average classification accuracies were 98% and 98.19% using the LDA and the logistic regression models, respectively. The STA/LTA algorithm was exploited as a pre-processing step to improve the classification accuracy of the proposed models. This algorithm served as adaptive thresholding for peak detection. The average achieved accuracy using the STA/LTA algorithm was improved to 99.17% and 99% using the LDA and logistic regression models, respectively. For future work, we will consider increasing the number of events to include human activities, such as walking, jumping, shaking, and climbing. Additionally, the number of FBG sensors can be increased to cover a longer perimeter. Another interesting plan would be enhancing the proposed models to simultaneously classify more than multiple events or measure the displacement of the fence. Moreover, to cover a longer perimeter or enhance the proposed model’s ability to classify more events simultaneously each with a different Bragg wavelength at different fence positions, one should modify the machine learning algorithm parameters to accommodate the new input features in the classification process. Furthermore, to sense dynamic events, one may use fast-scanning interrogation methods, such as using coherent receivers. We note that in real scenarios, one should use ML to distinguish between temperature and strain. One method to do so could be incorporating a network of FBGs on the same fence, where some sensors could be thermal FBGs [56] in order for the ML to be able to distinguish the different surrounding effects.

## Figures and Tables

**Table 1:** Labels of events to be classified.

Class	Label
No intruder	0
Intruder	1
Wind	2

**Table 2:** Fence metal properties.

Parameter	Value
Elongation %	22
Tensile Strength MPa	370
Yield Strength (0.2%) MPa	300
Shear Strength MPa	230
Hardness Brinell	100

**Table 3:** Features of the PXIe-4844 optical interrogator.

Parameters	Specification
Output power (continuous wave)	0.06 mW and 0.25 mW
Wavelength sweeping range	1510 to 1590 nm
Wavelength accuracy	1 pm
Dynamic range	40 dB
Hardware resolution	16 bit
Scan rate	10 Hz

**Table 4:** The strain-based optical FBG sensor specifications.

Parameters	Specification
Gage length	22 mm
Peak wavelength	1524.3 nm
Strain sensitivity	∼1.4 pm/μϵ
Peak reflectivity	>70%
FWHM	0.25 nm
Operating temperature range	−40 to $120{\;}^{\circ }$C
Strain limit	±2500μϵ

**Table 5:** The air blower specifications.

Parameters	Specification
Power	600 W
No load speed	Max of 16,000/min
Air volume	3.5 m${}^{3}$/min

## Data Availability

Not applicable.
